# In-Plane Compression Properties of Continuous Carbon-Fiber-Reinforced Composite Hybrid Lattice Structures by Additive Manufacturing

**DOI:** 10.3390/polym16131882

**Published:** 2024-07-01

**Authors:** Lingqi Jin, Jun Shi, Zhixin Chen, Zhiyang Wang, Yangfan Zhi, Lei Yang, Xinyi Xiao

**Affiliations:** 1 School of Transportation and Logistics Engineering, Wuhan University of Technology, Wuhan 430070, China; jlq@whut.edu.cn (L.J.); 18986621850@163.com (J.S.); czx001121@163.com (Z.C.); wangzhiyang@whut.edu.cn (Z.W.); zhiyangfan@whut.edu.cn (Y.Z.); 2 Department of Mechanical Engineering, The University of North Texas, Denton, TX 76203-1277, USA

**Keywords:** continuous carbon-fiber-reinforced composite, path arrangement, hybrid lattice structures, multiple permutations

## Abstract

Continuous-fiber-reinforced composite lattice structures (CFRCLSs) have garnered attention due to their lightweight and high-strength characteristics. Over the past two decades, many different topological structures including triangular, square, hexagonal, and circular units were investigated, and the basic mechanical responses of honeycomb structures under various load conditions, including tension, compression, buckling, shear, and fatigue were studied. To further improve the performance of the honeycombs, appropriate optimizations were also carried out. However, the mechanical properties of a single lattice often struggle to exceed the upper limit of its structure. This paper investigates the effect of permutation and hybrid mode on the mechanical properties of CFRCLSs by comparing five structures: rhomboid (R-type), octagon orthogonal array (OOA-type), octagon hypotenuse array (OHA-type), octagon nested array (ONA-type), and rhomboid circle (RC-type), with the conventional hexagonal structure (H-type). CFRCLS samples are fabricated using fused filament fabrication (FFF), with carbon-fiber-reinforced polylactic acid (PLA) as the matrix. The in-plane compression properties, energy absorption characteristics, and deformation behaviors of the hybrid structures were studied by experimental tests. The results demonstrate that different permutation and hybrid modes alter the deformation behaviors and mechanical properties of the structures. Taking elastic modulus as an example, the values of H-type, R-type, OOA-type, OHA-type, ONA-type, and RC-type are, respectively, 6.08 MPa, 5.76 MPa, 19.0 MPa, 10.3 MPa, 31.7 MPa, and 73.2 MPa, while the ratio of their masses is 1:1:1.10:1.52:1.66. Furthermore, hybrid lattice structures exhibit significantly improved mechanical properties compared to single lattice structures. Compared to the single structure R-type, the RC-type increases elastic modulus, yield strength, and energy absorption, respectively, by 12.7 times, 5.4 times, and 4.4 times.

## 1. Introduction

Fiber-reinforced composite materials are widely used in aerospace, marine engineering, advanced rail transit, new energy vehicles, biomedicine, electronic appliances, and many other fields for their superior mechanical properties [1,2,3,4,5], such as light weight [6], high strength [7], elasticity [8], and fatigue resistance [9].

Compared to normal structures, lattice structures, using the same amount of fiber, have higher specific strength and shock resistance, lower thermal conductivity, and better energy absorption [10,11,12]. However, due to the complex geometric characteristics of lattice structures, production is often limited by traditional processing methods. Challenges encountered in traditional fiber composite manufacturing encompass intricate processes, protracted production durations, and operational inconveniences associated with mold or cutting procedures. Additive manufacturing methods, based on the cumulative properties of materials, provide a technical approach for the rapid and efficient manufacturing of fiber-reinforced composite components [13,14,15]. Integrated forming is a significant advantage of additive manufacturing and has great potential for designing complex lattice structures [16]. It is one of the effective ways to achieve rapid, personalized, and complex structure manufacture of continuous-fiber-reinforced composite materials and break through the limitations of traditional manufacturing methods [17].

Additive manufacturing can sometimes result in inferior mechanical properties, making it crucial to find ways to enhance them [18]. There are many cell shapes in lattice structures such as tetrahedron, triangular, square, hexagonal, circle, and so on [19,20]. The review published by Qi et al. comprehensively summarized the mechanical properties of eight classical honeycombs, including re-entrant, double V-shaped, chiral, and star-shaped [21]. However, these classical honeycombs are not suitable for all practical applications. Structures with higher strength, better energy absorption capability, and wider Poisson’s ratio range are urgently needed for special applications. Based on these classical honeycombs, many advanced designs to improve the macro- and micromechanical properties of honeycomb materials should be proposed. In the past decades, many creative lattice structures have been studied [22,23]. Monkova et al. studied the effect of cell size and volume ratio of a body-centered cubic (BCC) lattice structure made from Acrylonitrile Butadiene Styrene (ABS) plastic [24]. Li et al. established an extended BCC lattice, by offsetting the body center position [25]. Alomar et al. proposed a lattice structure based on circular cells, where cells consist of identical circles along two vertical planes [26]. Zhang et al. designed a gradient honeycomb metastructure with optimization of the module stack large mutation genetic algorithms considering the material–structure–function integration, which can achieve the structural stealth for broadband microwave absorption [27]. Zhang et al. combined a vertically enhanced hourglass shape and a traditional hexagonal structure to design a novel hybrid structure, vertical strut, and hexagonal combined structures (VSHCSs) [28]. The VSHCS exhibits auxetic behavior, high plateau stresses, and excellent EAC owing to the unique design strategy of the hybrid unit cell. At the same time, the arrangement of the print path is also essential. Through the optimization of the path, the process defects caused by insufficient printing accuracy can be avoided. It can also guarantee the continuity and bonding strength of the fiber, improve the manufacturing accuracy, and shorten the printing time [29,30,31].

To date, most studies on lattice structures have primarily focused on the types of unit cells, with limited research on hybrid lattice structures composed of different unit cells [32]. The purpose of this paper is to combine different simple single cells and prove that hybrid structures have better mechanical properties at unit density and can obtain the structural advantages of their constituent cells. This means that by combining cells which have different property advantages, a structure with better overall performance can be obtained, and by adjusting the permutation and distribution of different cells, they will be more in line with the needs of practical applications. Therefore, this paper designs hybrid lattice structures and various octagon array structures to investigate the influence of hybrid cells and multiple permutations on mechanical and energy absorption properties. Although temperature is a key factor affecting mechanics, this paper mainly analyzes the performance at a normal temperature [33]. Additionally, all print paths are arranged based on Eulerian graph rules to ensure defect-free manufacturing processes.

## 2. Methodology

### 2.1. Materials and Manufacturing Method

**Figure 1:**
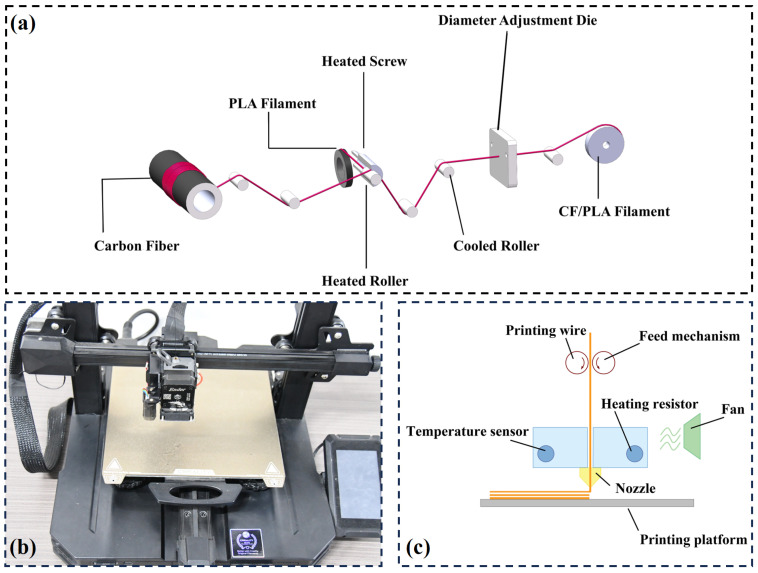
(**a**) Fused impregnation equipment; (**b**) Continuous fiber additive manufacturing equipment; (**c**) Processing diagram.

In this study, extrusion additive manufacturing (EAM) equipment was used to manufacture CFRCLS. Additive manufacturing of composite materials is mainly divided into in-situ impregnation process and pre-impregnation process [34], and this equipment is developed based on the latter one, as shown in Figure 1a. HTA40-E151K carbon fiber (the single filament diameter is 7 μm and the density is 1.78 g/cm^3^) was supplied by Dongguan Sovetl Special Rope & Webbing Co., Ltd. (Dongguan, China) The pre-impregnated material uses carbon-fiber-reinforced polylactic acid (PLA) as the matrix. The material density of the PLA was 1.31 g/cm^3^, and purchased from Shenzhen Creality 3D Technology Co., Ltd. (Shenzhen, China). The pre-impregnated filament is produced by self-designed fused impregnation equipment with a diameter of 0.45 mm and a fiber volume content of 19.1%.

As shown in Figure 1b, the pre-impregnated filament is stored in the reel at first, and then pulled by the feed mechanism during printing. When the filament is through the heating tube, it is fused, extruded from the nozzle, and deposited onto the printing platform. The printing path is a complete loop. After the single-layer printing, the nozzle lifts a layer height. Repeat in this way. The processing diagram is shown in Figure 1c.

### 2.2. The Design of Lattice Structures

Ha et al. discovered that the mechanical properties of circular structures can be enhanced when inserting smaller circular tubes between four circular tubes. The mean compressive stress increased nearly 2 times while the thickness t=0.5 mm [35]. The traditional lattice structures usually have a 45-degree inclined fracture zone [36]. Therefore, we designed hybrid structures based on the rules that increase oblique supports. The structure design idea is as follows: The array of octagonal cells will create different regions. So, when combining the circle and diamond cells to rearrange its array, it becomes a hybrid structure.

**Figure 2:**
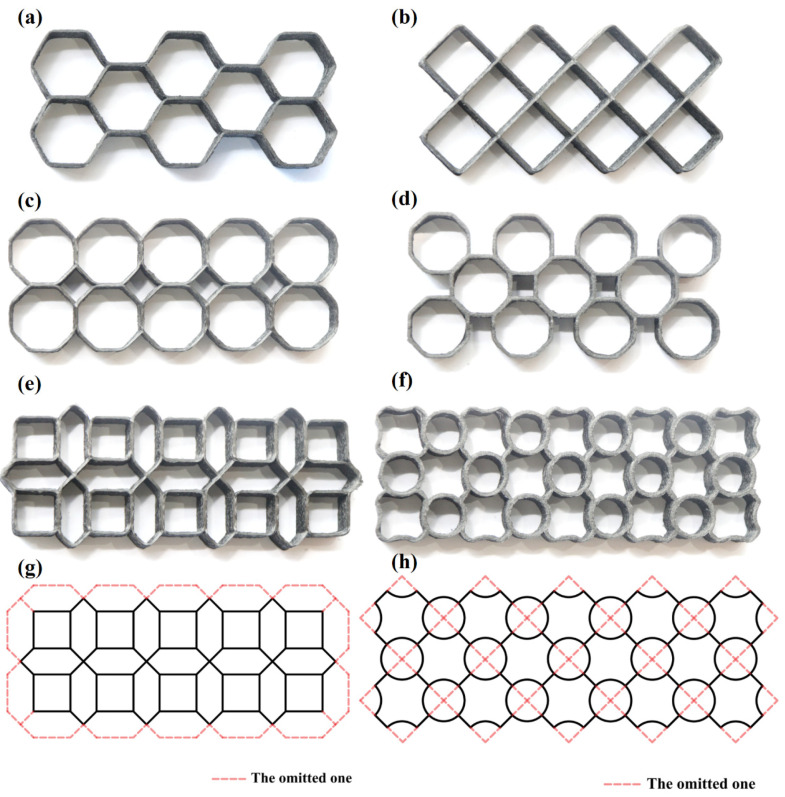
Pictures of printed samples: (**a**) H-type; (**b**) R-type; (**c**) OOA-type; (**d**) OHA-type; (**e**) ONA-type; (**f**) RC-type, and structure formation diagram of (**g**) ONA-type; (**h**) RC-type.

Six types of samples were designed and are shown in Figure 2. H-type is composed of hexagonal cells. R-type is composed of rhomboid cells. OOA-type consists of octagonal cells connected by their horizontal and vertical sides pair in pair, with a diamond space in the middle of the structures. OHA-type is composed of octagonal cells connected by hypotenuse pair in pair, and a square space is formed in the middle of the structures. ONA-type is obtained by nesting octagonal cells and several lines are omitted, as shown in Figure 2g. RC-type is composed of the same rhomboid cells as R-type and middle circle cells, as shown in Figure 2h.

The forming parameters including the thickness of layers $t_{z}$, printing speed *v*, the platform temperature $T_{1}$, and the nozzle temperature $T_{2}$, will influence the properties of samples [37]. This study’s parameters are shown in Table 1, and the related physical quantities of the samples are shown in Table 2.

### 2.3. Path Arrangement

During the additive manufacturing process of CFRCLS, if the filament is cut off while printing, it can result in empty travels and disrupt the continuity of the fibers, thereby affecting the strength of the structure. Moreover, the influence of tension force can cause fiber misalignment at locations with closed angles, leading to reduced printing accuracy or failure. Additionally, if no cross is generated at the path joints, the tension of the continuous fiber may cause premature turning, resulting in insufficient fiber adhesion at the joint [38]. These issues highlight the importance of resolving them during path planning.

To achieve high-quality CFRCLS manufacturing, certain constraints must be applied to the printing paths. To ensure continuity, it is desirable for the printing path for each layer to form a loop [39]. This loop structure allows the starting point of each layer to remain consistent, thus avoiding shearing and empty travels during the printing process. Additionally, it is necessary to print every edge in the structure diagram at least once to create a complete structure.

**Figure 3:**
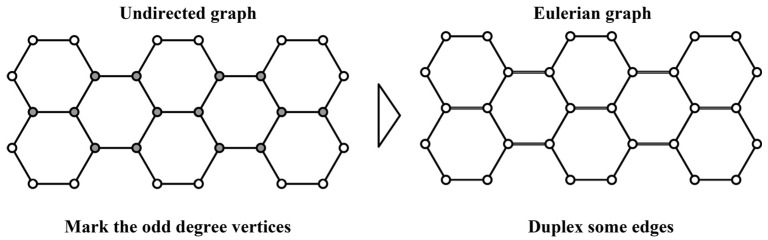
The schematic diagram that an undirected graph transforms into an Eulerian circuit.

**Figure 4:**
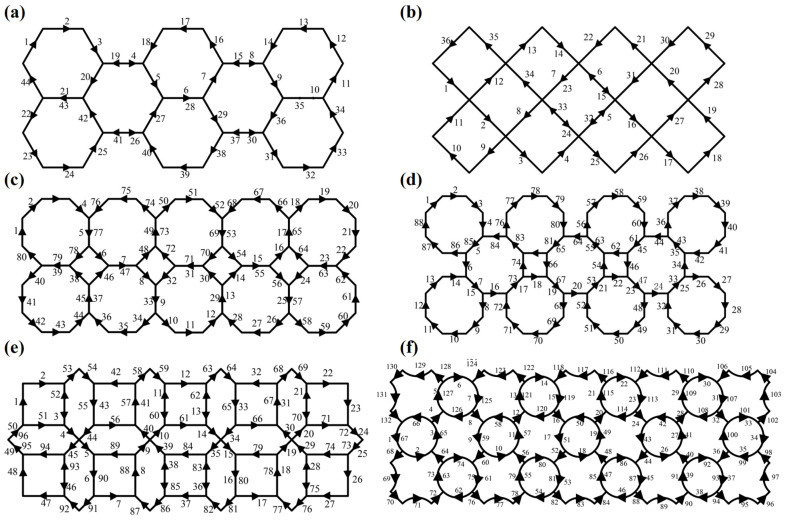
The designed paths of lattice structures: (**a**) H-type; (**b**) R-type; (**c**) OOA-type; (**d**) OHA-type; (**e**) ONA-type; (**f**) RC-type.

Based on the two goals mentioned above, we need some mathematical knowledge related to graph theory. This allows us to simplify the structure graph into an Eulerian graph that meets all the manufacturing goals [40]. However, not all structure graphs can be directly simplified into Eulerian graphs. The sufficient and necessary conditions to become an Eulerian graph are: (1) The graph must be a loop. (2) All vertices in the graph must have an even degree (the degree of a vertex is the number of edges connected to it). Therefore, for an undirected graph, we first need to determine whether it meets the sufficient and necessary conditions of an Eulerian graph. If it does, we can obtain an Eulerian circuit directly from this graph. If not, we need to double some edges of the undirected graph, i.e., print these edges twice per layer, to obtain an Eulerian circuit, as shown in Figure 3. Finally, an Eulerian circuit is chosen to avoid sharp turns and ensure that all the joints are crossed in the final printing path. The complete printing paths for all structures are illustrated in Figure 4.

### 2.4. Test Equipment and Experimental Settings

**Figure 5:**
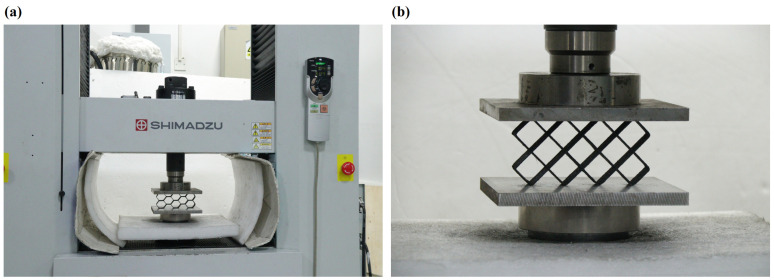
Experimental equipment (**a**) and installation of sample (**b**).

SHIMADZU AG-IC 100 KN (Tokyo, Japan) testing machine was utilized to carry out in-plane compression tests. The sample was positioned between the two square blocks. The lower one is fixed on the testing machine, and the upper one is movable. During the test, the upper block moved downward at a speed of 2.96 mm/min and applied load force until the densification. At the same time, a camera device was placed outside the testing machine to capture the deformation behavior of the samples during the compression process. The experimental equipment and installation of the sample are depicted in Figure 5.

In the process of in-plane compression, the nominal stress and nominal strain of the structure are calculated as follows:

(1) $$\sigma =\frac{F}{A_{t}}$$

(2) $$\varepsilon_{y}=\frac{\Delta_{y}}{H}$$

where *F* is the applied load. $A_{t}$ is the vertical initial cross-sectional area, i.e., $A_{t}=L\times t$. Δ_*y*_ is the compression displacement. *H* is the height of the sample.

## 3. Results and Discussions

### 3.1. Quasi-Static Compressive Responses and Mechanical Properties

**Figure 6:**
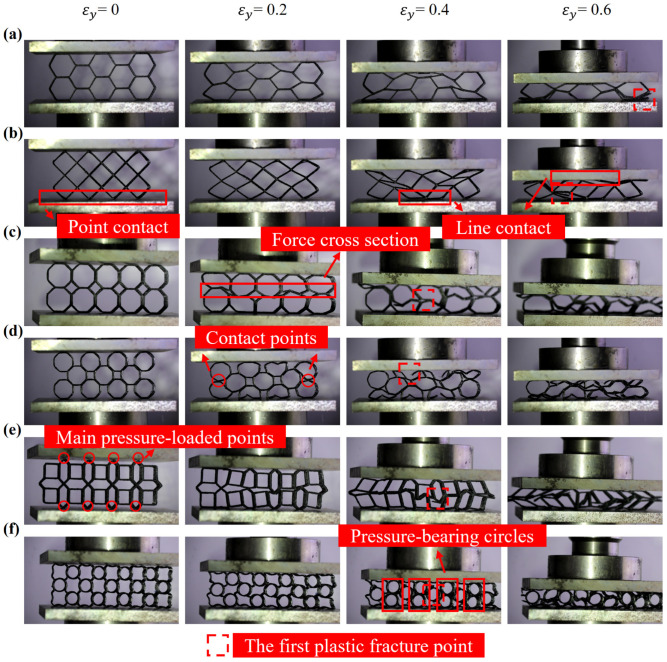
The fracture collapse process of lattice structures: (**a**) H-type; (**b**) R-type; (**c**) OOA-type; (**d**) OHA-type; (**e**) ONA-type; (**f**) RC-type.

**Figure 7:**
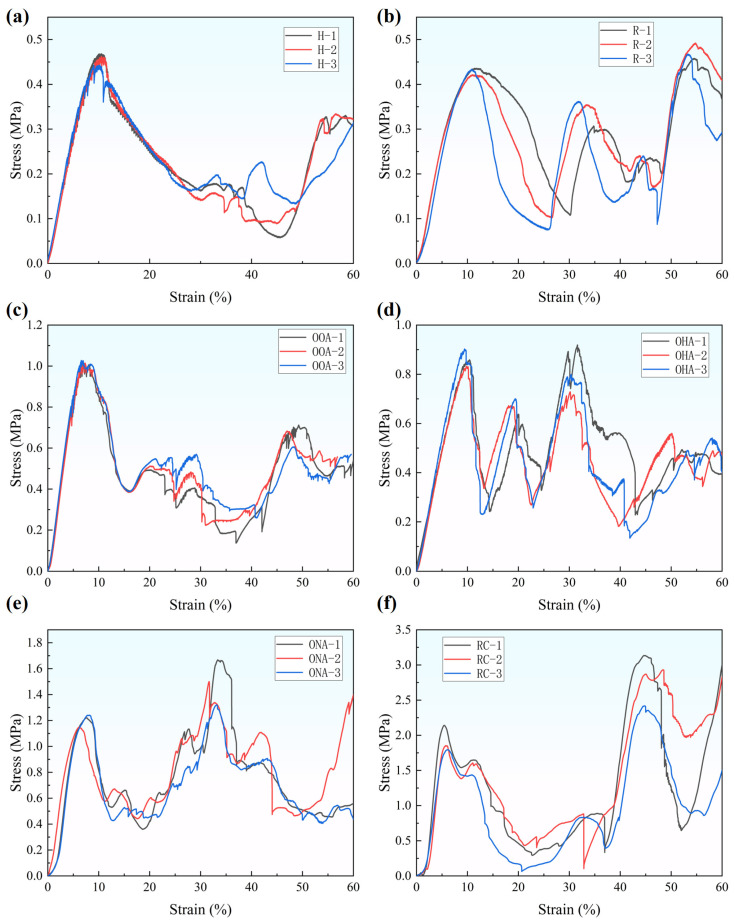
The stress–strain curves of lattice structures: (**a**) H-type; (**b**) R-type; (**c**) OOA-type; (**d**) OHA-type; (**e**) ONA-type; (**f**) RC-type.

Figure 6 and Figure 7 illustrate the static compression response of H-type, R-type, OOA-type, OHA-type, ONA-type, and RC-type structures. The mechanical curves of multiple specimens have high repeatability before $\varepsilon_{y}=0.2$. However, due to the large unit cell size, low filling rate, and little support, there is no obvious plateau in the stress–strain curve, as mentioned by Li et al. [41], but multiple waveforms with large changes appear until they enter the densification stage. With the stress–strain curves, we can analyze the deformation behaviors of structures [42].

H-type’s deformation behavior is classical. After a short period of overall deformation, the structure begins to collapse cell by cell so the curve shown in Figure 7a drops after the first peak (i.e., the elastic limit). When $\varepsilon_{y}=0.5$, the completely collapsed cells become thick boards and support other intact cells, corresponding to another crest in the curve. Then, the cells in the oblique direction collapse first, and the collapsed cell walls end up with an inverted V shape; the R-type structure also deforms very quickly. The cells in the V-shaped direction begin to collapse first, and, finally, plastic fractures appear. It is worth noting that the contact form between the structure and the loading blocks is point contact at first, and with the deformation and collapse of the cells, the upper and lower surfaces, successively, become line contact, as shown in Figure 6b. Line contact can bear more load so the curve shown in Figure 7b has an extra two-wave crest; when the OOA-type is compressed, cells in the same layer are evenly stressed. The boundary between the layers becomes tortuous first, as shown in Figure 6c, then most cells collapse quickly and a large number of plastic fractures occur. Its curve trends and formation mechanisms are similar to H-type’s: in the fracture collapse process of the OHA-type, the upper and lower layers of cells squeeze each other to fill the middle gap of the structure, resulting in a tendency to oblique deformation of the cells. 

The curve in Figure 7d has a small crest between strain equals 1.5 and 2.5, because the left and right cells in the upper and lower layers touch and support each other in the mesosphere, as shown in Figure 6d. Cells in the lower layer changed shape first, followed by the upper layer. The upper layer, as the loading end, is the first to fracture. This process corresponds to the third crest in the curve; the ONA-type has relatively strong resistance to deformation. The stress is borne first by the points, as shown in Figure 6e, and then the vertical hexagon unit cells begin to deform and tilt. The square unit cells touch the loading block and the whole structure shares the load, which is reflected in the stress–strain curve as a second peak’s stress exceeds the maximum stress in the elastic stage, as shown in Figure 7e. After that, the structure collapses and the plastic fracture occurs. That is the reason why the curve comes into a low plateau stage when $\varepsilon_{y}=0.6$; the RC-type goes through a long period of overall deformation. When $\varepsilon_{y}=0.4$, the side supports belonging to the rhomboid part are deformed and fractured, and the circular cell begins to bear the main pressure so that the value of the second wave crest is much larger than the first peak, as shown in Figure 6f and Figure 7f. Whether it is the first half or the second half of the fracture collapse process, its pressure resistance is far more than other structures. And the curve surges when strain equals 0.6, because the RC-type enters the densification stage.

**Figure 8:**
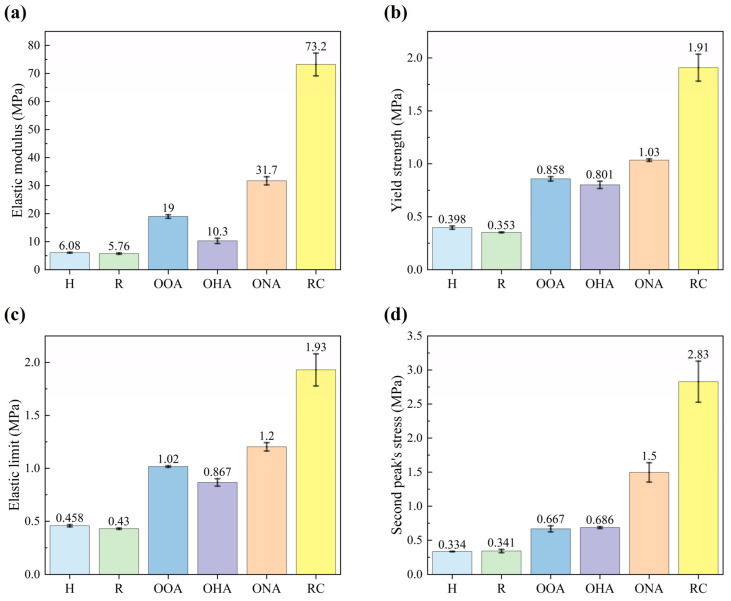
The obtained mechanical properties of structures, including (**a**) Elastic modulus, (**b**) Yield strength, (**c**) Elastic limit, (**d**) Second peak’s stress.

The mechanical properties of the samples can be obtained according to the stress–strain curve. The obtained mechanical properties of the structures are presented in Figure 8. The size relationship of each parameter of the samples is similar, and the elastic modulus most obviously shows the difference. Take the elastic modulus as an example: the values of the H-type, R-type, OOA-type, OHA-type, ONA-type, and RC-type are, respectively, 6.08 MPa, 5.76 MPa, 19.0 MPa, 10.3 MPa, 31.7 MPa, and 73.2 MPa, while their masses are, respectively, 12.4 g, 12.4 g, 13.6 g, 12.9 g, 18.8 g, and 20.6 g. Preliminary analysis can be obtained: the H-type and R-type have the worst properties, the octagon arrays have relatively better properties, and the RC-type has the best.

To be specific, the elastic modulus and yield strength of the H-type are 6.08 MPa and 0.398 MPa, respectively, which are very similar to 5.76 MPa and 0.353 MPa, respectively, for the R-type. Each parameter other than the elastic modulus of the OHA-type is nearly twice that of the H-type, such as the yield strength of 0.801 MPa. The OOA-type’s properties are a little better than the OHA-type’s, and its elastic modulus is 1.9 times more than the OHA-type’s. The elastic modulus, yield strength, and elastic limit of the ONA-type are, respectively, 31.7 MPa, 1.03 MPa, and 1.2 MPa, while, respectively, 73.2 MPa, 1.91 MPa, and 1.93 MPa for the RC-type. This is far more than 19 MPa, 0.858 MPa, and 1.02 MPa for the OOA-type, which has the best mechanical properties among the experimented single structures.

Comparing the mechanical properties of octagon arrays, for example: the ratio of the elastic modulus of the OOA-type, OHA-type, and ONA-type is 1.84:1:3.08, while the ratio of their masses is only 1.05:1:1.46. The result shows the influence of permutation that the orthogonal array has better mechanical properties than the hypotenuse array, and the nested array to a certain extent transforms the single lattice structure into the hybrid lattice structure. Another hybrid lattice structure, the RC-type, increased elastic modulus by nearly 12.7 times more than the R-type, while the ratio of their masses is only 1.66:1. Its properties are dramatically higher than other single lattice structures. The comparison in Figure 8c,d shows that the second peak’s stress of the single lattice structure is always lower than the elastic limit (i.e., the first peak’s stress), but hybrid lattice structures are not. For example, the elastic limit and second peak’s stress are 1.2 MPa and 1.5 MPa, respectively, for the ONA-type, and 1.93 MPa and 2.83 MPa, respectively, for the RC-type. This is because a hybrid lattice structure always consists of the base unit cells and the core unit cells. The core unit cells can not only enhance the load capacity of the base unit cells, but also bear the main load after the base unit cells are collapsed, contributing to a higher peak of stress value.

### 3.2. Energy Absorption Ability

**Figure 9:**
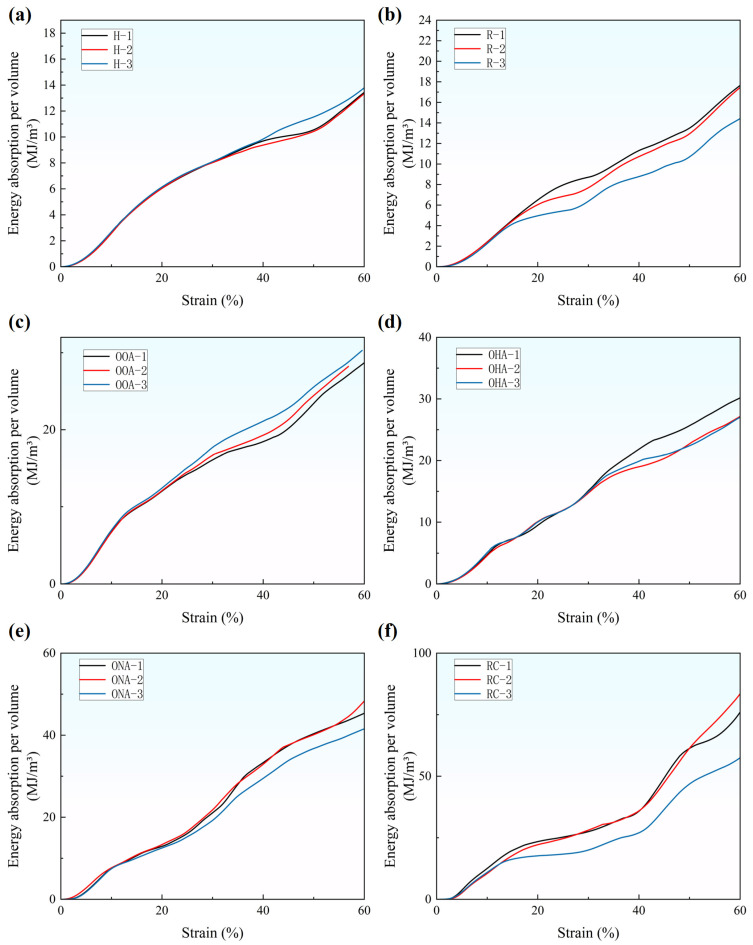
Energy absorption per volume of lattice structures: (**a**) H-type; (**b**) R-type; (**c**) OOA-type; (**d**) OHA-type; (**e**) ONA-type; (**f**) RC-type.

The cumulative energy absorption values per unit volume can be calculated by [43],

(3) $$w_{v}=\int_{0}^{\varepsilon }\sigma (\varepsilon )d\varepsilon$$

and are shown in Figure 9. The average energy absorption values per volume of the H-type, OOA-type, OHA-type, ONA-type, R-type, RC-type, are respectively, 13.7, 29.4, 28.6, 45.1, 15.8, and 71.8 ${MJ/m}^{3}$ when the strain is 0.6. The energy absorption curves are all close to the linear relationship, except for the RC-type. Its curve’s slope changes greatly at the point of ε=0.2 and ε=0.4, which corresponds to the time when the rhomboid cells begin to collapse and when the circle cells begin to bear load, as shown in Figure 6f and Figure 9f.

**Figure 10:**
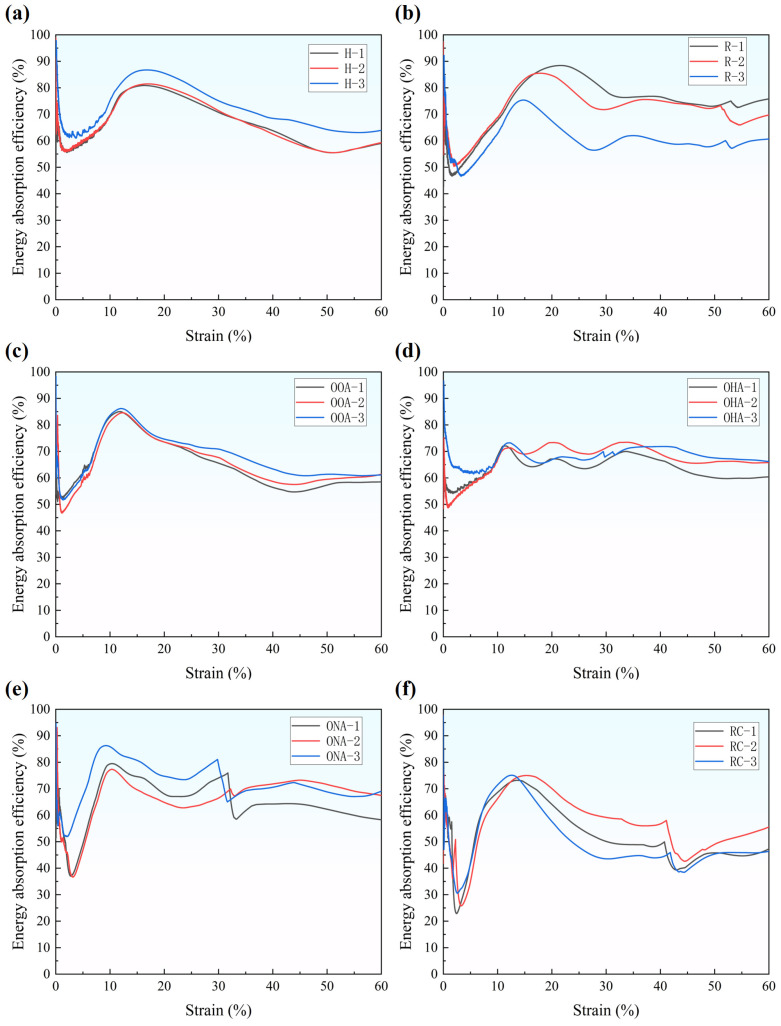
Energy absorption efficiency of lattice structures: (**a**) H-type; (**b**) R-type; (**c**) OOA-type; (**d**) OHA-type; (**e**) ONA-type; (**f**) RC-type.

Figure 10 presents the energy absorption efficiency, defined as the ratio of energy absorption to the product of the maximum stress and strain, elaborating the energy absorption capability of designed lattice structures [43,44]. The efficiency curves of the H-type, R-type, and OOA-type all have a process of a rapid rise and then a gradual decline. They also have a maximum not lower than 80% and a minimum not lower than 50%. Different from other single lattice structures, the OHA-type has a very steady efficiency curve with a value of 65%, and does not have an obvious peak, as shown in Figure 10d. The efficiency values of the OHA-type and RC-type are relatively low and fluctuate more greatly. Especially for the RC-type, the lowest value of the curve reaches 25%, and the efficiency is less than 50% at the strain between 30% and 40%.

To conclude, the hybrid lattice structure can absorb more energy because the core unit cells can give support to the base unit cells and increase both energy absorption abilities. However, the hybrid lattice structure will have lower energy absorption efficiency and it changes more frequently. Besides, the efficiency curve of the hypotenuse array is more steady, which means wasting less energy under high-strain conditions.

We also use specific energy absorption $S^{\prime }$ and mean crushing force $M^{\prime }$ to study energy absorption characteristics [45]. Specific energy absorption represents the amount of energy absorbed per unit mass of a lattice structure.

**Figure 11:**
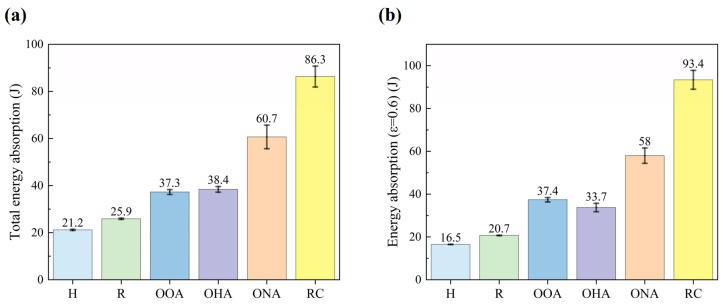
The energy absorption before the densification point (**a**) and the energy absorption when ε=0.6 (**b**).

The definition formula is as follows:

(4) $$S^{\prime }=\frac{E_{a}}{m}$$

where $E_{a}$ is the total energy absorption of the structure during the compression process and *m* is the mass of the structure. *E_a_* and $E_{\varepsilon =0.6}$ are shown in Figure 11, and can be calculated as follows:

(5) $$E_{a}=\int_{0}^{y_{d}}Fdy$$

(6) $$E_{a}=\int_{0}^{y_{\varepsilon =0.6}}Fdy$$

where *y* stands for compressive displacement, *F* for load, and $y_{d}$ represents the displacement when the sample enters the densification stage.

The mean crushing force $M^{\prime }$ is calculated as follows:

(7) $$M^{\prime }=\frac{1}{y_{d}}E_{a}$$

The other parameters mentioned above are shown in Table 3.

The $S^{\prime }$ and $M^{\prime }$ of the structures have similar size relationships with mechanical properties and energy absorption. Taking the mean crushing force $M^{\prime }$ as an example, the values of the H-type, R-type, OOA-type, OHA-type, ONA-type, and RC-type are 0.54 KN, 0.64 KN, 1.16 KN, 1.06 KN, 1.92 KN, and 3.06 KN, respectively. Comparing the $\varepsilon_{d}$ of structures, a trend is that the higher the fiber filling rate, the earlier it is to enter the densification stage, and the better energy absorption ability. However, the OOA-type has an abnormally low $\varepsilon_{d}$ of 0.61, only higher than the RC-type of 0.57, because its unit cells’ main support direction is parallel to the load direction. The result shows that the hybrid lattice structure greatly increases the energy absorption ability but comes into the densification stage earlier. Furthermore, the orthogonal array can bring the densification stage forward and absorb more energy at high strain.

## 4. Conclusions

In this paper, five different continuous-fiber-reinforced composite lattice structures (CFRCLS) were designed and fabricated via the extrusion additive manufacturing (EAM) technique. The mechanical properties, energy absorption ability, and deformation behaviors of the hybrid structures were investigated under in-plane compression experiments. The main conclusions can be drawn as follows:

(1) Permutation and hybrid mode of lattice structures will influence the mechanical properties and energy absorption abilities. The size relationship of each parameter is similar, and the elastic modulus is influenced the most.
(2) Multiple permutations have different characteristics of the energy absorption process and mechanical properties. The hypotenuse array and the orthogonal array both have high energy absorption efficiency. Although having similar parameters, the orthogonal array has a great enhancement of elastic modulus compared to the hypotenuse array.
(3) The RC-type, while greatly reducing the filling rate of traditional circular structures, still has mechanical properties and energy absorption ability far exceeding that of single lattice structures such as hexagonal structures.
(4) Hybrid lattice structures can significantly enhance the structure’s properties and exceed single lattice structures’ limit. They can also have more balanced properties compared to their constituent cells. It is also worth noting that a hybrid lattice structure will have lower energy absorption efficiency.

This study investigated the significant mechanical advantages of the hybrid lattice structure and the effect of cell permutation, which could provide a reference on how to enhance and change the properties of lattice structures. Combining unit cells which have different structural advantages, we can create more balanced and comprehensive hybrid lattice structures. And the inference is put forward here: by adjusting various ratios and volume fractions of the different unit cells in a hybrid structure, the properties of the structure can be changed to any value within a certain range, to meet the needs of practical applications.

## Figures and Tables

**Table 1:** Parameters of printing process.

$t_{z}$/mm	*n*	$T_{1}$/°C	$T_{2}$/°C	$v/\mathrm{mm}\cdot {min}^{-1}$
0.2	100	45	210	90

Notes: *n* is the number of layers.

**Table 2:** Actual geometric parameters and masses of the lattice structures.

Types	*L*/mm	*H*/mm	*t*/mm	*m*/g
H	119.00	52.85	19.62	12.4
R	110.51	54.75	19.50	12.4
OOA	121.34	49.32	19.90	13.6
OHA	114.40	53.37	19.81	12.9
ONA	127.60	50.15	20.10	18.8
RC	129.17	45.20	20.05	20.6

Notes: *L*, *H*, *t*, and *m* are the actual width, height, mass, and thickness of the lattice structures.

**Table 3:** $S^{\prime }$, $M^{\prime }$, $\varepsilon_{d}$, and $y_{d}$ of structures.

Types	$S^{\prime }/J\cdot g^{-1}$	$M^{\prime }$/KN	$\varepsilon_{d}$	$y_{d}$/mm
H	1.71	0.54	0.75	39.38
R	2.09	0.64	0.74	40.36
OOA	2.74	1.16	0.61	32.13
OHA	2.98	1.06	0.68	36.16
ONA	3.23	1.92	0.63	31.51
RC	4.19	3.60	0.57	26.03

Notes: $\varepsilon_{d}$ and $y_{d}$ are the densification strain and densification displacement, respectively, of the lattice structures.

## Data Availability

Data are contained within the article.

## References

[1] Balakrishnan P., John M.J., Pothen L., Sreekala M., Thomas S. (2016). Natural fibre and polymer matrix composites and their applications in aerospace engineering. Advanced Composite Materials for Aerospace Engineering.

[2] Mallick P.K. (2007). Fiber-Reinforced Composites: Materials, Manufacturing, and Design.

[3] Rajak D.K., Pagar D.D., Menezes P.L., Linul E. (2019). Fiber-reinforced polymer composites: Manufacturing, properties, and applications. Polymers.

[4] Seydibeyoglu M.O., Mohanty A.K., Misra M. (2017). Fiber Technology for Fiber-Reinforced Composites.

[5] Yashas Gowda T., Sanjay M., Subrahmanya Bhat K., Madhu P., Senthamaraikannan P., Yogesha B. (2018). Polymer matrix-natural fiber composites: An overview. Cogent Eng..

[6] Cheng P., Peng Y., Li S., Rao Y., Le Duigou A., Wang K., Ahzi S. (2023). 3D printed continuous fiber reinforced composite lightweight structures: A review and outlook. Compos. Part B Eng..

[7] Jayan J.S., Appukuttan S., Wilson R., Joseph K., George G., Oksman K. (2021). An introduction to fiber reinforced composite materials. Fiber Reinforced Composites.

[8] Whitney J., Riley M. (1966). Elastic properties of fiber reinforced composite materials. AIAA J..

[9] Ansari M.T.A., Singh K.K., Azam M.S. (2018). Fatigue damage analysis of fiber-reinforced polymer composites—A review. J. Reinf. Plast. Compos..

[10] Helou M., Kara S. (2018). Design, analysis and manufacturing of lattice structures: An overview. Int. J. Comput. Integr. Manuf..

[11] Maconachie T., Leary M., Lozanovski B., Zhang X., Qian M., Faruque O., Brandt M. (2019). SLM lattice structures: Properties, performance, applications and challenges. Mater. Des..

[12] Pan C., Han Y., Lu J. (2020). Design and optimization of lattice structures: A review. Appl. Sci..

[13] Beaman J., Bourell D.L., Seepersad C., Kovar D. (2020). Additive manufacturing review: Early past to current practice. J. Manuf. Sci. Eng..

[14] Plocher J., Panesar A. (2019). Review on design and structural optimisation in additive manufacturing: Towards next-generation lightweight structures. Mater. Des..

[15] Yang L., Zhou L., Lin Y., Hu Y., Yan C., Shi Y. (2024). Failure mode analysis and prediction model of additively manufactured continuous carbon fiber-reinforced polylactic acid. Polym. Compos..

[16] Hu Y., Lin Y., Yang L., Wu S., Tang D., Yan C., Shi Y. (2024). Additive manufacturing of carbon fiber-reinforced composites: A review. Appl. Compos. Mater..

[17] Parandoush P., Lin D. (2017). A review on additive manufacturing of polymer-fiber composites. Compos. Struct..

[18] Chacón J.M., Caminero M.A., García-Plaza E., Núnez P.J. (2017). Additive manufacturing of PLA structures using fused deposition modelling: Effect of process parameters on mechanical properties and their optimal selection. Mater. Des..

[19] Gibson L.J. (2003). Cellular solids. MRS Bull..

[20] Zhang Q., Yang X., Li P., Huang G., Feng S., Shen C., Han B., Zhang X., Jin F., Xu F. (2015). Bioinspired engineering of honeycomb structure–Using nature to inspire human innovation. Prog. Mater. Sci..

[21] Qi C., Jiang F., Yang S. (2021). Advanced honeycomb designs for improving mechanical properties: A review. Compos. Part B Eng..

[22] Tao W., Leu M.C. Design of lattice structure for additive manufacturing. Proceedings of the 2016 International Symposium on Flexible Automation (ISFA).

[23] Jin J., Wu S., Yang L., Zhang C., Li Y., Cai C., Yan C., Shi Y. (2024). Ni–Ti multicell interlacing Gyroid lattice structures with ultra-high hyperelastic response fabricated by laser powder bed fusion. Int. J. Mach. Tools Manuf..

[24] Monkova K., Vasina M., Zaludek M., Monka P.P., Tkac J. (2021). Mechanical vibration damping and compression properties of a lattice structure. Materials.

[25] Li P., Yang F., Bian Y., Zhang S., Wang L. (2021). Deformation pattern classification and energy absorption optimization of the eccentric body centered cubic lattice structures. Int. J. Mech. Sci..

[26] Alomar Z., Concli F. (2021). Compressive behavior assessment of a newly developed circular cell-based lattice structure. Mater. Des..

[27] Zhang Y., Dong H., Yu C., Wang Z., Huang Y. (2024). Metastructure based broadband structural stealth with material-structure-function integration. Compos. Sci. Technol..

[28] Zhang X., Hao H., Tian R., Xue Q., Guan H., Yang X. (2022). Quasi-static compression and dynamic crushing behaviors of novel hybrid re-entrant auxetic metamaterials with enhanced energy-absorption. Compos. Struct..

[29] Jimbo K., Tateno T. (2022). Design optimization of infill pattern structure and continuous fiber path for CFRP-AM: Simultaneous optimization of topology and fiber arrangement for minimum material cost. Precis. Eng..

[30] Jin Y., He Y., Fu G., Zhang A., Du J. (2017). A non-retraction path planning approach for extrusion-based additive manufacturing. Robot. Comput.-Integr. Manuf..

[31] Sugiyama K., Matsuzaki R., Malakhov A.V., Polilov A.N., Ueda M., Todoroki A., Hirano Y. (2020). 3D printing of optimized composites with variable fiber volume fraction and stiffness using continuous fiber. Compos. Sci. Technol..

[32] Park K.-M., Min K.-S., Roh Y.-S. (2021). Design optimization of lattice structures under compression: Study of unit cell types and cell arrangements. Materials.

[33] Mu Y., Jin Y., Ji H., Luo J., Li G., Xu M., Li H., Deng B., Du J. (2024). Mechanical performance of interpenetrating phase composites with multi-sheet lattice structures. Int. J. Mech. Sci..

[34] Pandelidi C., Bateman S., Piegert S., Hoehner R., Kelbassa I., Brandt M. (2021). The technology of continuous fibre-reinforced polymers: A review on extrusion additive manufacturing methods. Int. J. Adv. Manuf. Technol..

[35] San Ha N., Pham T.M., Tran T.T., Hao H., Lu G. (2022). Mechanical properties and energy absorption of bio-inspired hierarchical circular honeycomb. Compos. Part B Eng..

[36] Yang L., Wu S., Yan C., Chen P., Zhang L., Han C., Cai C., Wen S., Zhou Y., Shi Y. (2021). Fatigue properties of Ti-6Al-4V Gyroid graded lattice structures fabricated by laser powder bed fusion with lateral loading. Addit. Manuf..

[37] Kristiawan R.B., Imaduddin F., Ariawan D., Ubaidillah A.Z. (2021). A review on the fused deposition modeling (FDM) 3D printing: Filament processing, materials, and printing parameters. Open Eng..

[38] Wang K., Wang D., Liu Y., Gao H., Yang C., Peng Y. (2023). Path Planning and Bending Behaviors of 3D Printed Continuous Carbon Fiber Reinforced Polymer Honeycomb Structures. Polymers.

[39] Ding D., Pan Z., Cuiuri D., Li H. (2015). A practical path planning methodology for wire and arc additive manufacturing of thin-walled structures. Robot. Comput.-Integr. Manuf..

[40] Yamamoto K., Luces J.V.S., Shirasu K., Hoshikawa Y., Okabe T., Hirata Y. (2022). A novel single-stroke path planning algorithm for 3D printers using continuous carbon fiber reinforced thermoplastics. Addit. Manuf..

[41] Li Q., Magkiriadis I., Harrigan J.J. (2006). Compressive strain at the onset of densification of cellular solids. J. Cell. Plast..

[42] Hyer M.W., White S.R. (2009). Stress Analysis of Fiber-Reinforced Composite Materials.

[43] Yang L., Li Y., Wu S., Chen P., Wu H., Su J., Wang H., Liu J., Yan C., Shi Y. (2022). Tailorable and predictable mechanical responses of additive manufactured TPMS lattices with graded structures. Mater. Sci. Eng. A.

[44] Song J., Wang Y., Zhou W., Fan R., Yu B., Lu Y., Li L. (2019). Topology optimization-guided lattice composites and their mechanical characterizations. Compos. Part B Eng..

[45] Zhang Y., Xu X., Wang J., Chen T., Wang C.H. (2018). Crushing analysis for novel bio-inspired hierarchical circular structures subjected to axial load. Int. J. Mech. Sci..

